# Micro-morphologic changes around biophysically-stimulated titanium implants in ovariectomized rats

**DOI:** 10.1186/1746-160X-3-28

**Published:** 2007-07-16

**Authors:** Kivanc Akca, Ebru Sarac, Ugur Baysal, Mete Fanuscu, Ting-Ling Chang, Murat Cehreli

**Affiliations:** 1 Research Scholar, Division of Restorative Dentistry, The Jane and Jerry Weintraub Center for Reconstructive Biotechnology, UCLAc School of Dentistry, USA; 2 Associate Professor, Department of Prosthodontics, Faculty of Dentistry, Hacettepe University, Ankara, Turkey; 3 Private Practice, Obstetrics and Gynecology, Ankara, Turkey; 4 Associate Professor, Department of Electrical and Electronics Engineering, Hacettepe University, Ankara, Turkey; 5 Associate Clinical Professor, Division of Restorative Dentistry, The Jane and Jerry Weintraub Center for Reconstructive Biotechnology, UCLA School of Dentistry, USA; 6 Associate Clinicial Professor, Division of Advanced Prosthodontics, The Jane and Jerry Weintraub Center for Reconstructive Biotechnology, UCLA School of Dentistry, USA; 7 Associate Professor of Prosthodontics, CosmORAL Oral and Dental Health Polyclinics, Ankara, Turkey

## Abstract

**Background:**

Osteoporosis may present a risk factor in achievement of osseointegration because of its impact on bone remodeling properties of skeletal phsiology. The purpose of this study was to evaluate micro-morphological changes in bone around titanium implants exposed to mechanical and electrical-energy in osteoporotic rats.

**Methods:**

Fifteen 12-week old sprague-dowley rats were ovariectomized to develop osteoporosis. After 8 weeks of healing period, two titanium implants were bilaterally placed in the proximal metaphyses of tibia. The animals were randomly divided into a control group and biophysically-stimulated two test groups with five animals in each group. In the first test group, a pulsed electromagnetic field (PEMF) stimulation was administrated at a 0.2 mT 4 h/day, whereas the second group received low-magnitude high-frequency mechanical vibration (MECHVIB) at 50 Hz 14 min/day. Following completion of two week treatment period, all animals were sacrificed. Bone sites including implants were sectioned, removed *en bloc *and analyzed using a microCT unit. Relative bone volume and bone micro-structural parameters were evaluated for 144 μm wide peri-implant volume of interest (VOI).

**Results:**

Mean relative bone volume in the peri-implant VOI around implants PEMF and MECHVIB was significantly higher than of those in control (*P *< .05). Differences in trabecular-thickness and -separation around implants in all groups were similar (*P *> .05) while the difference in trabecular-number among test and control groups was significant in all VOIs (*P *< .05).

**Conclusion:**

Biophysical stimulation remarkably enhances bone volume around titanium implants placed in osteoporotic rats. Low-magnitude high-frequency MECHVIB is more effective than PEMF on bone healing in terms of relative bone volume.

## Background

Implant designs and treatment protocols are continuously evolving to promote osseointegration and the clinical success of oral implants. While many oral implant systems have been designed thus far to ameliorate biologic host response, biomechanical needs, which essentially dominate functioning has not been profoundly recognized [1]. To date, some methods of applying biophysical stimuli to implants such as electrical stimulation [2-4], pulsed electromagnetic fields (PEMF) [5-7], and mechanical vibration (MECHVIB) [8] have been tested for promotion of fracture healing and tissue differentiation at the bone-implant interface [9,10]. It is unfortunate that these experimental approaches have not been used to derive any therapeutic instrument coupled with an application schedule for oral/orthopedic implants so far.

The application of static and pulsed magnetic fields have been demonstrated to promote bone formation at sites of injury, such as fracture [11]. In the context of implants, magnetic fields under 10 mT have been shown to increase cell attachment and proliferation on titanium implant surfaces [12]. A study in the rat femur also showed that application of PEMF increased bone contact ratios of implants and that the applied dose had a critical role on bone apposition [7]. Under serum-free conditions, mechanical stimulation by intermittent hydrostatic compression has been demonstrated to increase sulfate content mineralization of calcifying cartilage of fetal long bone rudiments [13]. The application of MECHVIB have shown that daily 1 Hz/100-sec regimen led to 28% bone ingrowth increase and at 20 Hz, the amount of ingrowth increased to 69% [8]. In addition, another study demonstrated that the intraosseous stability of implants subjected to direct 3 Hz mechanical vibration with a force of 5 N for 1800 cycli for 6 weeks were improved [14] Indeed, application of low-amplitude, high frequency mechanical stimuli seems very attractive from a therapeutic point of view to promote osseointegration [15], as the anabolic effects of low-magnitude mechanical signals have already been demonstrated on bone [16]. Further the risk of soft and hard tissue damage will be avoided at such low amplitudes, and the noninvasive and nonpharmacologic nature of the technique could improve the well-being of the patients during treatment. Nevertheless, current limitations of these approaches, i.e., long application sessions for PEMF and possible direct mechanical stimulation of implants via intraoral mechanical-shaking devices do not virtually seem feasible.

Osteoporosis leads to decrease in bone density and bone-implant contact ratio of implants. A study has shown that transmitted MECHVIB could increase density of load-bearing bones in osteoporotic women [17]. In search of ways to promote histodynamics of tissue differentiation and biomechanical potential of oral implants in osteoporotic women, it was hypothesized that using transmitted MECHVIB could facilitate application of biophysical stimuli to the critical area [17,18] and its user-friendly nature could potentially be used for therapeutic application. In addition, it was assumed that the outcome of MECHVIB could be superior to PEMF in terms of bone response. The purpose of this study was, therefore, to compare micro-morphologic changes in bone around implants subjected to PEMF and transmitted (indirect) low-magnitude high-frequency MECHVIB in osteoporotic rats.

## Materials and methods

### Animals, care and ovariectomy

The experiments were undertaken in 15 locally-bred 12-week old Sprague-Dowley female rats. The animals were cared for according to the policies and principles established by the Animal Welfare Act and the NIH Guide for Care and Use of Laboratory Animals (publication # 86–23). The surgical and experimental protocols for the animals were approved by the ethical committee of the animal research facility of Hacettepe University (2004/45-9). During the entire test period, the animals were kept in rooms illuminated from 07:00 to 19:00 hours (12 h light/12 h dark cycle), maintained at 21–23°C, and had full access to low-calcium (0.1 %) powdered diet, prepared according to AIN-93M prescription, and water *ad libitum*. All surgical procedures were performed under general anesthesia using a mixture of ketamin (Ketalar, Parke-Davis; 50 mg/kg i.m.) and xylazine (Rompun, Bayer; 15 mg/kg i.m.). Following surgery, each animal was kept in a 25°C incubator until it regained consciousness.

During bilateral ovariectomy, the hair at the back of the anesthetized animals was shaved and the skin disinfected with 70% ethanol. The overiectomy of the animals were undertaken by an experienced gynecologist. A dorsal midline incision was made through the skin at the level of both kidneys. The exposed ovaries through the thin muscle wall by retracting the skin laterally toward either side were pulled into the incision and excised after the ligation of the upper horn of the uterus [19,20]. Following completion of surgery, facia and skin were sutured in layers. Before the experiments and after 4 weeks of recovery and adaptation period, induction of osteoporosis was verified by measuring the level of serum alkaline phosphatase (ALP) in the blood collected randomly from 5 animals (Table 1). An increase in ALP values was accepted as induction of osteoporosis [21]. Quantative determination of ALP was undertaken by regular serum ALP biochemical analyses using ALP liquid acc to IFCC using Roche/Hitachi 904/911/912/9217/MOD P/D: ACN 158 Analyzer. The test principle is based on colorimetric assay in accordance with a standardized method:

**Table 1:** ALP levels (U/L) before and after ovariectomy in randomly selected 5 animals.

Animal	BO	AO
#1	72	285
#2	76	305
#3	87	298
#4	78	312
#5	85	264

BO: Before ovariectomy; AO: after ovariectomy

1. Sample and addition of 2-Amino-2-methyl-1-propanol: 1.12 mol/L, pH 10.44 (30°C);magnesium acetate: 2.49 mmol/L; zinc sulfate: 0.50 mmol/L; N-(2-hydroxyethyl)-ethylenediamine triacetic acid: 2.49 mmol/L

2. Addition of p-Nitrophenyl phosphate: 99.5 mmol/L; pH 8.50 (25°C); preservatives.

p-nitrophenyl phosphate+H2O→ALPphosphate+p nitrophenol
 MathType@MTEF@5@5@+=feaafiart1ev1aaatCvAUfKttLearuWrP9MDH5MBPbIqV92AaeXatLxBI9gBaebbnrfifHhDYfgasaacH8akY=wiFfYdH8Gipec8Eeeu0xXdbba9frFj0=OqFfea0dXdd9vqai=hGuQ8kuc9pgc9s8qqaq=dirpe0xb9q8qiLsFr0=vr0=vr0dc8meaabaqaciaacaGaaeqabaqabeGadaaakeaacqqGWbaCcqqGTaqlcqqGUbGBcqqGPbqAcqqG0baDcqqGYbGCcqqGVbWBcqqGWbaCcqqGObaAcqqGLbqzcqqGUbGBcqqG5bqEcqqGSbaBcqqGGaaicqqGWbaCcqqGObaAcqqGVbWBcqqGZbWCcqqGWbaCcqqGObaAcqqGHbqycqqG0baDcqqGLbqzcqGHRaWkcqqGibasdaWgaaWcbaGaeGOmaidabeaakiabb+eapnaaoqcaleaacqqGbbqqcqqGmbatcqqGqbauaeqakiaawkziaiabbchaWjabbIgaOjabb+gaVjabbohaZjabbchaWjabbIgaOjabbggaHjabbsha0jabbwgaLjabgUcaRiabbchaWjabbccaGiabb6gaUjabbMgaPjabbsha0jabbkhaYjabb+gaVjabbchaWjabbIgaOjabbwgaLjabb6gaUjabb+gaVjabbYgaSbaa@731C@

In the presence of magnesium and zinc ions, p-nitrophenyl phosphate is cleaved by phosphates into phosphate and p nitrophenol. The p nitrophenol is released proportional to the ALP activity and is measured photometrically.

### Implants and surgery

A total of 30 cylindrical implants (Ø 1 mm × 5 mm) were obtained from a commercially-pure titanium rod (99.6%; Goodfellow Cambridge Ltd., Huntingdon, England). The implants were washed in ultrasonic deionized water, then further in trichloroethylene (99.5 %) and ethanol (70%), and sterilized before tests [22]. During surgery, both cortices of the tibia were perforated with low rotational speed under constant saline cooling with a surgical drill having a diameter smaller than the implant's diameter. The rationale behind this approach was to achieve good primary stability of the cylindrical implants. Two implants were placed bilaterally placed in the proximal metaphyses of tibia. The flaps were closed with resorbable sutures (Vicryls 3-0, Ethicon GmbH, Norderstadt, Germany) and left to heal for 1 week.

### Test groups and application of biophysical stimuli

**Figure 1:**
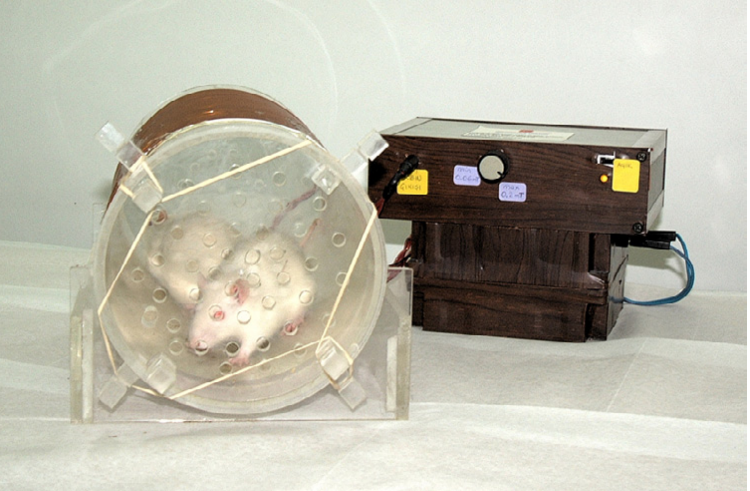
Fabricated custom-made device to deliver PEMF stimulation on osteoporotic rats.

**Figure 2:**
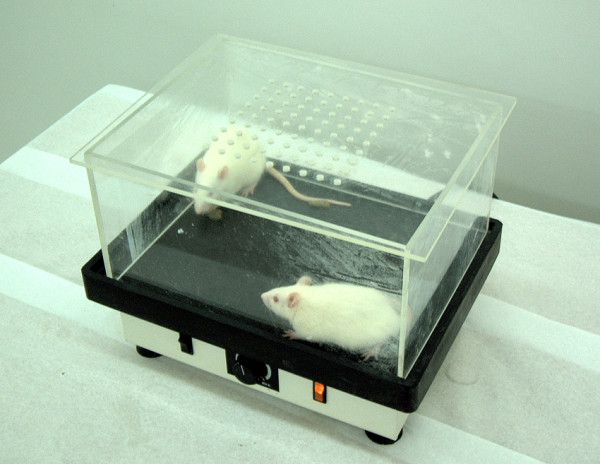
Plexiglass bordered vibrating plate used to administer mechanical low-magnitude high-frequency to osteoporotic rats.

Upon placement of the implants, the animals were randomly divided into three groups. Group 1 served as control. In Group 2, PEMF stimulation was administrated at 0.2 mT 4 h/day for the implants [7] (Fig. 1). The custom-made PEMF delivery device was fabricated at the Department of Electrical and Electronics Engineering of Hacettepe University and tested for accuracy using hall effect gauss/tesla meter (Sypris F.W. Bell Model 5080, Florida, USA) having 1% accuracy in measurement range. In Group 3, low-magnitude high-frequency MECHVIB at 5 N/50 Hz 14 min/day was applied to the implants, while each animal was set on a mechanical vibrating plate (Vibratore Shaker 6, Carlo Degiorgi, Milano, Italy) with plexiglass borders to keep the test animal within the test zone during the therapeutic stimulation period (Fig. 2). The mechanical vibrating plate provides a barely perceptible stimulus, which does not alter animal behavior. This application allowed a ground-based whole body application through the hindfeets of the animal contacting the vibrating plate [17,18]. In addition, this technique allows the animal to move freely on the vibrating plate [18]. After 14 days, all animals were sacrificed and the tibia of each animal was removed *en bloc *and kept in physiologic saline maintained at 21–23°C.

### Micro-morphologic evaluation of bone around implants

**Figure 3:**
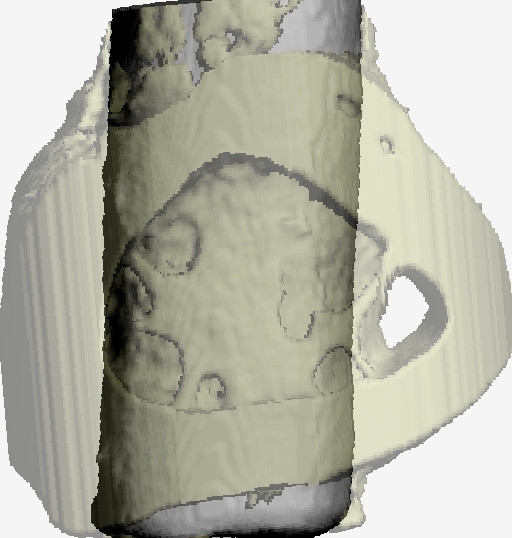
3a and 3b. 3-D microCT view of a titanium implant (a) and illustration of defined peri-implant VOIs (b).)

Each specimen was subjected to micro-tomographic scanning [23] using desktop MicroCT (μCT40, ScancoMedical, Bassersdorf, Switzerland) with a resolution of 16 × 16 × 16 μm^3^. The specimen were scanned with up to 320 transverse slices, where each slice consisted of 1024 × 1024 pixels and followed by off-line reconstruction. Prior to micro-morphologic analyses, transverse slices cervically and apically resting in cortical bone were discarded. Three longitudinal volume of interest (VOI) each with 48 μm-thick (3 voxels × 16 μm resolution) were nominally defined, and consecutively numbered 1 to 3 starting from the implant surface (Fig. 3). The resulting images were then segmented by using different thresholds for bone and implant [24]. The specific thresholds for titanium and bone were determined by superimposing segmented over original grayscale images.

The relative bone volume (BV/TV: %) and micro-morphometric bone parameters including trabecular thickness (Tb.Th: mm), trabecular separation (Tb.Sp: mm) and trabecular number (Tb.N: 1/mm) were separately calculated for each VOI. Morphometric indices, which can be directly determined from the binarized VOI, are the bone volume density (BV/TV), which is a measure of the volume of the bone trabeculae relative to the total volume of the VOI, and the trabecular number (Tb.N). From these directly determined indices, other indices are derived such as the trabecular thickness (Tb.Th), the trabecular separation (Tb.Sp) [25]. The correlation between structural parameters obtained by micro-CT and conventional histomorphometry has been investigated to determine which micro-CT parameter is most closely related to osteoporotic fracture [26]. It has been ascertained that BV/TV, 3D-Tb.Th and 3D-Tb.N were correlated with those values on conventional histomorphometry. Therefore, trabecular bone parameters gained from micro-CT scanning in this study seems to provide valuable information.

### Statistical analysis

The data of each test group were compared with one-way analysis of variance (ANOVA) at a confidence level set at 95% and further with Post Hoc Tests (LSD) at 95% to determine different groups.

## Results

**Figure 4:**
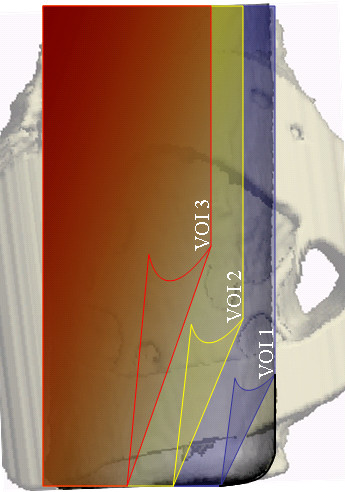
3-D microCT view of VOI 1–3 (from left to right) around control (top), PEMF-stimulated (middle), and MECHVIB-stimulated (bottom) implants.

3-D microCT view of three VOIs around control, PEMF-stimulated, and MechV-stimulated implants are presented in Fig 4. Descriptive statistics of BV/TV, Tb.Th and Tb.Sp, and Tb.N are presented in Tables 2, 3, 4, 5 and Post Hoc Test (LSD) comparisons between groups are shown in Table 6. ANOVA of BV/TV revealed a significant difference among test and control groups (p = 0.000) in vicinities VOI-1, VOI-2 and VOI-3. The peri-implant relative bone volume around MECHVIB-stimulated implants were higher than control and PEMF-stimulated implants (p = 0.000), whereas similar values were obtained for the latter two groups (*P *> .05) (Table 2). ANOVA of morphologic evaluations revealed that Tb.Th and Tb.Sp around implants in all groups were similar (*P *> .05) (Table 3 and 4, respectively). The difference in Tb.N among test and control groups was significant in all VOIs (*P *< .05). Tb.N around MECHVIB-stimulated implants was higher than control and PEMF-stimulated implants (*P *< .05), and similar around control and PEMF-stimulated implants (*P *> .05) in all VOIs (Table 5).

**Table 2:** ANOVA of peri-implant relative bone volume (BV/TV: %) around implants of test and control groups (n = 15).

	p	Group	Mean	Std. Deviation	Std. Error	95% Confidence interval for mean		Minimum	*Maximum*
								
						Lower Bound	Upper Bound		
VOI-1	0.000	Control	4.0	0.002	0.0007	0.002	0.006		
		MECHVIB	15.5	0.005	0.001	0.113	0.196	0.083	*0.225*
		PEMF	7.0	0.002	0.0007	0.005	0.009	0.049	*0.123*
VOI-2	0.000	Control	22.4	0.008	0.002	0.162	0.286	0.121	*0.370*
		MECHVIB	61.4	0.216	0.006	0.459	0.769	0.358	*0.950*
		PEMF	34.3	0.007	0.002	0.287	0.339	0.220	*0.509*
VOI-3	0.000	Control	17.0	0.005	0.001	0.128	0.211	0.084	*0.265*
		MECHVIB	47.8	0.20	0.006	0.334	0.621	0.246	*0.793*
		*PEMF*	*26.5*	*0.008*	*0.002*	*0.207*	*0.322*	*0.180*	*0.447*

**Table 3:** ANOVA of trabecular-thickness (mm) around implants of test and control groups (n = 15).

	p	Group	Mean	Std. Deviation	Std. Error	95% Confidence interval for mean		Minimum	*Maximum*
								
						Lower Bound	Upper Bound		
VOI-1	0.997	Control	0.140	0.005	0.001	0.100	0.180	0.080	*0.226*
		MECHVIB	0.141	0.002	0.008	0.121	0.161	0.105	*0.178*
		PEMF	0.142	0.005	0.001	0.104	0.179	0.090	*0.260*
VOI-2	0.655	Control	0.130	0.004	0.002	0.009	0.165	0.072	*0.218*
		MECHVIB	0.147	0.002	0.009	0.125	0.168	0.105	*0.192*
		PEMF	0.133	0.004	0.001	0.100	0.166	0.082	*0.236*
VOI-3	0.752	Control	0.188	0.005	0.002	0.149	0.227	0.114	*0.288*
		MECHVIB	0.198	0.005	0.002	0.162	0.234	0.133	*0.278*
		*PEMF*	*0.180*	*0.005*	*0.002*	*0.139*	*0.220*	*0.110*	*0.296*

**Table 4:** Descriptive statistics of trabecular-separation (mm) around implants of test and control groups (n = 15).

	p	Group	Mean	Std. Deviation	Std. Error	95% Confidence interval for mean		Minimum	*Maximum*
								
						Lower Bound	Upper Bound		
VOI-1	0.161	Control	0.546	0.254	0.008	0.363	0.728	0.253	*0.938*
		MECHVIB	0.530	0.221	0.007	0.372	0.688	0.106	*0.821*
		PEMF	0.718	0.229	0.007	0.554	0.882	0.402	*1.065*
VOI-2	0.134	Control	0.572	0.249	0.007	0.393	0.750	0.303	*0.918*
		MECHVIB	0.521	0.204	0.006	0.375	0.668	0.122	*0.803*
		PEMF	0.725	0.225	0.007	0.563	0.886	0.412	*1.046*
VOI-3	0.216	Control	0.573	0.204	0.006	0.426	0.720	0.365	*0.861*
		MECHVIB	0.474	0.168	0.005	0.354	0.595	0.190	*0.735*
		*PEMF*	*0.634*	*0.222*	*0.007*	*0.475*	*0.793*	*0.334*	*0.950*

**Table 5:** ANOVA of trabecular-number (1/mm) around implants of test and control groups (n = 15).

	p	Group	Mean	Std. Deviation	Std. Error	95% Confidence interval for mean		Minimum	*Maximum*
								
						Lower Bound	Upper Bound		
VOI-1	0.003	Control	2.027	0.830	0.262	1.432	2.621	1.305	*3.884*
		MECHVIB	3.170	0.734	0.244	2.615	3.744	1.535	*4.181*
		PEMF	2.09	0.636	0.201	1.642	2.552	1.083	*3.162*
VOI-2	0.025	Control	2.214	0.786	0.248	1.651	2.777	1.413	*3.846*
		MECHVIB	3.014	0.716	0.226	2.502	3.527	1.730	*3.622*
		PEMF	2.170	0.676	0.213	1.687	2.654	1.176	*3.381*
VOI-3	0.006	Control	2.051	0.576	0.182	1.638	2.463	1.479	*3.259*
		MECHVIB	2.803	0.406	0.128	2.512	3.094	1.826	*3.234*
		*PEMF*	*2.166*	*0.527*	*0.166*	*1.788*	*2.543*	*1.258*	*2.996*

**Table 6:** Post Hoc Test (LSD) comparisons between groups.

	BV/TV			Tb.N		
	
	VOI-1	VOI-2	VOI-3	VOI-1	VOI-2	VOI-3
Control-MECHVIB	0.00	0.00	0.00	0.002	0.021	0.003
Control-PEMF	0.117	0.071	0.113	0.832	0.894	0.617
MECHVIB-PEMF	0.00	0.00	0.001	0.004	0.015	0.009

## Discussion

The present study was designed to gain insight into micro-morphologic changes in bone around biophysically-stimulated implants in an animal study. In the present study, the rationale behind using ovariectomized rats was to explore the effect of MECHVIB and PEMF stimulation on bone micromorphology around titanium implants placed in metabolically compromised bone condition in terms of the possible worst case in bone to undertake the study [27]. Unlike previous studies [8] low-magnitude high-frequency MECHVIB was delivered using a "transmitted" approach, as this could facilitate administration of mechanical signals in a user-friendly nature and improve patients comfort. Indeed, the results of the present study show that low-magnitude high-frequency MECHVIB could transmit through bone [17], reach the implant, and improve osteogenic response in the vicinity of implants. The increased peri-implant relative bone volume (BV/TV) around MECHVIB-stimulated implants clearly shows that a transmitted application of 5 N/50 Hz for 14 min/day for 2 weeks improves trabecular bone, which may essentially refer to improvement of implant anchorage and stability. Moreover, it is well-known that mechanical signals have a pronounced influence on the development and differentiation of mesenchymal tissues [28] and that the magnitude, frequency, and the rate of experienced strain appear as important interrelated determinants of skeletal differentiation. Therefore, the regimen of MECHVIB used in the present study, probably fall into "species-independent" band of low-amplitude strains (< 500 με) [8] in bone that act as a "growth factor"[28]. Since very small magnitudes of physiologic strains have been shown to increase bone mineral content and induce osteogenesis [8,11], and the direction of bending and axial loading does not have any effect on course of remodelling [29], artificial loading of implants by means of transmitted MECHVIB could potentially ameliorate bone-implant interface at early stages of function [15].

PEMF have been demonstrated to promote bone ingrowth into titanium and hydroxyapatite-coated implants, but not into tricalcium phosphate implants [6,7,30]. However, the outcome of transmitted application of 5 N/50 Hz for 14 min/day being higher than PEMF-stimulation implies that micro-morphologic properties of bone around implants is not higher than MECHVIB by administration of 0.2 mT 4 h/day PEMF [7]. Nevertheless, we should note that bone response to different doses of PEMF could lead to different results. Because it was not the core of the present study to explore the most osteogenic dose of PEMF, a dose of 0.2 mT 4 h/day, which had been shown to increase bone contact ratio and bone area ratio around implants were used [7]. Moreover, the duration of application seems as an important factor for bone response [6] and long application sessions for PEMF in the context of stimulating implants could inherently make the technique unpleasant for the patient, regardless of the dose administered. Therefore, not only the bone structural response but also the nature of PEMF seems rather weak in comparison to transmitted MECHVIB.

MicroCT with a limit of approximately 10 μm, provides the best resolution and has therefore become the imaging modality of choice for evaluation of trabecular bone in research over the past decade [25]. Using microCT, the interrelationship of mechanical and microstructural properties of trabecular bone is important for better understanding the consequences of trabecular remodeling. In the present study, increased peri-implant relative bone volume (BV/TV) and higher trabecular number were found around MECHVIB-stimulated implants, which imply that the stiffness of the tissue in the vicinity of the implants had increased. However, MECHVIB was unable to increase trabecular thickness and/or decrease trabecular separation. Consequently, trabecular thickness and separation around implants in all groups was similar. A reduction of trabecular thickness or increase in trabecular separation could have indicated decrease in mechanical properties of bone around the implants. The lack of increase in trabecular thickness and separation in test groups could be related to the dose administered, the duration of the experiment or both, but do not necessarily refer to the weakness of the techniques used to administer biophysical stimuli. Further studies are required to gain insight into structural and biomechanical characterization of bone around biophysically-stimulated implants.

## Authors' contributions

The study design was established by Murat Cehreli and Kývanc Akca, who also wrote the manuscript. The ovariectomy surgeries were undertaken by Ebru Sarac. The animal experiments were performed by Kývanc Akca. The PEMF apparatus was designed and fabricated by Ugur Baysal. Mete Fanuscu and Ting-Ling Chang performed the microfocus CT analysis.
